# FSCN1 promotes proliferation, invasion and glycolysis via the IRF4/AKT signaling pathway in oral squamous cell carcinoma

**DOI:** 10.1186/s12903-023-03191-9

**Published:** 2023-07-25

**Authors:** Liang Li, Lihui Chen, Zhangwei Li, Shiqin Huang, Yaoyao Chen, Zhiyong Li, Wenkuan Chen

**Affiliations:** 1 Department of Stomatology, the First Affiliated Hospital of Guangdong Pharmaceutical University, Guangzhou, 510080 China; 2 State Key Laboratory of Oncology in South China, Sun Yat-sen University Cancer Center, Guangzhou, 510060 China

**Keywords:** FSCN1, IRF4, AKT, OSCC, Proliferation, Invasion, Glycolysis

## Abstract

**Background:**

Oral squamous cell carcinoma (OSCC) is a disease with increasing incidence worldwide that leads to deformity and death. In OSCC, fascin actin-bundling protein 1 (FSCN1) is an oncogene involved in the tumorigenesis process. However, the functions and potential mechanisms of FSCN1 in the OSCC tumorigenesis process have not been reported thus far.

**Methods:**

We used qRT‒PCR to detect the expression of FSCN1 in 40 paired OSCC tumor tissues (tumor) and neighboring noncancerous tissues. The role of FSCN1 was also assessed in vitro through colony formation, CCK-8, and transwell assays. Moreover, glucose consumption was detected. Western blotting was used to confirm the interaction of FSCN1, IRF4 and AKT.

**Results:**

FSCN1 was remarkably overexpressed in OSCC tissues and cell lines compared to corresponding controls. In addition, colony formation, CCK-8, and transwell assays revealed a notable reduction in OSCC growth and invasion when FSCN1 was silenced. FSCN1 silencing remarkably suppressed OSCC glycolysis. Mechanistic studies showed that FSCN1 achieves its function partially by activating interferon regulatory factor 4 (IRF4) and the AKT pathway in OSCC.

**Conclusion:**

In conclusion, our study investigated the functions and mechanisms of the FSCN1/IRF4/AKT pathway in OSCC progression. In OSCC, FSCN1 is likely to be a biomarker and therapeutic target.

**Supplementary Information:**

The online version contains supplementary material available at 10.1186/s12903-023-03191-9.

## Introduction

Oral squamous cell carcinoma (OSCC) is a disease with increasing incidence worldwide that leads to deformity and death [1]. Recently, the incidence and mortality rates of OSCC are increasing worldwide, and this increase in mortality rate is associated with regional and distant metastasis, which leads to a worse prognosis [2]. Despite improvements in OSCC diagnosis and treatment, almost half of patients with OSCC die within five years of diagnosis [3]. Improving our understanding of the complex process of OSCC could provide additional opportunities to improve treatment and patient prognosis.

Fascin actin-bundling protein 1 (FSCN1) belongs to the fascin family of proteins that bind to actin [4]. FSCN1 serves as an important regulator in cell migration and cell-to-cell interactions [5, 6]. Overexpression of FSCN1, which promotes cell migration, has been reported to be associated with cancer metastasis in many studies [7]. For example, in ovarian cancer, FSCN1 promotes epithelial-mesenchymal transition (EMT) by interacting with and increasing the expression levels of snail1 [8]. In hepatocellular carcinoma, FSCN1 is overexpressed, and by promoting EMT, FSCN1 increases the chemoresistance capacity of hepatocellular carcinoma [9].

In OSCC, FSCN1 is identified as an oncogene involved in the tumorigenesis process. Amplification and mRNA upregulation of FSCN1 was found in OSCC [10]. In addition, FSCN1 expression levels were related to the prognosis of patients with OSCC, and patients with high FSCN1 expression had poorer outcomes than patients with low FSCN1 expression [11]. There are, however, no reports on the functions and potential mechanisms of FSCN1 in OSCC tumorigenesis.

In this study, we aimed to examine the expression patterns of FSCN1 in OSCC cell lines and tissues as well as its biological functions in processes including proliferation, invasion, and glycolysis. Moreover, we investigated the underlying mechanisms by which FSCN1 functions in the OSCC tumorigenesis process, which should shed some light on the potential of FSCN1 as a diagnostic biomarker and therapeutic target molecule for OSCC.

## Materials and methods

### Cell culture

Human normal oral keratinocytes (HNOK) and human OSCC cell lines (SCC9, SCC15, SCC25, HN4, HSC3, HSC6 and CAL27) were purchased from ATCC (USA) and maintained at 37 °C and 5% CO_2_. Cells were grown in DMEM (HyClone) supplemented with 10% FBS (Gibco). Mycoplasma infection and cell line authenticity were verified by DNA fingerprinting before use.

### Tissue specimens

Forty paired OSCC tissue specimens (Tumor) and neighboring noncancerous tissue specimens (Normal) were obtained during surgery at Sun Yat-sen University Cancer Center. Fresh tissues were stored in TRIzol (Invitrogen, USA) immediately after being cut and were used for subsequent qRT‒PCR assays. In accordance with its ethical approval, this study was conducted at Sun Yat-sen University Cancer Center and was carried out in accordance with the Declaration of Helsinki. Written informed consent was provided by all patients.

### qRT‒PCR

Total OSCC cell and tissue RNA was obtained by TRIzol (Invitrogen). With the SYBR Premix Ex Taq kit (Takara, China), mRNA expression was detected on an Applied Biosystems 7500 Fast Real-Time PCR System. The following primers were synthesized by Life Technologies: FSCN1 forward, 5’-CCAGGGTATGGACCTGTCTG-3’, reverse, 5’-GTGTGGGTACGGAAGGCAC-3’; GAPDH forward, 5’-ACAACTTTGGTATCGTGGAAGG-3’, reverse, 5’-GCCATCACGCCACAGTTTC-3’. The relative gene expression levels were calculated with the 2^−ΔΔCT^ formula with GAPDH as the internal reference.

### Cell transfection

Briefly, si-FSCN1#1 (5’-GCGCCUACAACAUCAAAGA-3’), si-FSCN1#2 (5’-GCCCAUGAUAGUAGCUUCA-3’), si-FSCN1#3 (5’-GCAGCCUGAAGAAGAAGCA-3’) or the appropriate control si-NC (GeneCopoeia, USA) was transiently transfected into the SCC15 and HSC3 cell lines (Lipofectamine 3000, Invitrogen). The cells were collected and subjected to further experiments 48 h later.

### ShRNA construction and lentiviral infection

GeneCopoeia synthesized FSCN1 shRNAs, and the Lenti-PacTM HIV Expression Packaging Kit (GeneCopoeia) was used to generate lentiviruses expressing sh-FSCN1. SCC15 and HSC3 cell lines were infected with lentiviruses using Lipo3000. SCC15-sh-FSCN1 and HSC3-sh-FSCN1 cells were selected with puromycin (2 µg/mL) and subjected to further experiments.

### CCK-8 assay

SCC15-sh-FSCN1 cells, HSC3-sh-FSCN1 cells and control cells were seeded in 96-well plates (3000 cells/well). Forty-eight hours later, 10 µl CCK-8 solution (Dojindo, Japan) was added. Finally, the absorbance at 450 nm was detected 2 h later using a microplate reader.

### Colony formation assay

SCC15-sh-FSCN1 cells, HSC3-sh-FSCN1 cells and control cells were seeded in six-well plates (1000 cells/well). Fourteen days later, methanol was used to fix the colonies for 30 min, and crystal violet (0.1%) was used to stain them for 30 min. The number of colonies was counted and averaged in three independent experiments.

### Transwell assay

SCC15-sh-FSCN1 cells, HSC3-sh-FSCN1 cells and control cells were resuspended in serum-free medium and seeded into 24-well transwell chambers (1 × 10^4^ cells/well) precoated with Matrigel (BD Bioscience, USA) for the invasion assay. A 20% FBS solution was added to the lower chamber, while medium without FBS was added to the upper chamber. After 24 h of incubation, the invaded cells were fixed with cold methanol, and crystal violet (0.1%) was used to stain the invaded cells for 20 min. The number of invaded cells was quantified in five randomly selected fields under a light microscope.

### Glycolytic metabolism detection

Glucose consumption was detected by the Glucose Test Kit (Biovision, USA). SCC15-sh-FSCN1 cells, HSC3-sh-FSCN1 cells and control cells were seeded (1 × 10^6^ cells/well each), and glucose concentration reduction was detected with cell culture medium. Similarly, lactate production was investigated with the Lactate Assay Kit (Biovision). The ATP/ADP ratio was detected by the ApoSENSOR ADP/ATP Ratio Assay Kit (Biovision) [12].

### Western blotting

Total OSCC cell proteins were extracted with PMSF and RIPA lysis buffer (Sigma, USA), and the protein concentration was determined with a Pierce BCA protein assay kit. Proteins were subjected to 12% SDS‒PAGE and transferred onto a PVDF membrane. The membrane was blocked with 5% skim milk powder for 2 h at room temperature. Then, primary antibodies against the following proteins were added for incubation overnight at 4 °C: FSCN1 (1:1000, #ab126772, Abcam, USA), IRF4 (1:500, #sc-6059, Santa Cruz Biotechnology, USA), AKT (1:500, #9272, CST, USA), p-AKT (1:500, #9271, CST), E-cadherin (1:1000, #3195, CST), Vimentin (1:1000, #5741, CST) and GAPDH (1:1000, #AF7021, Affinity, USA). Next, the membrane was incubated with HRP-linked secondary antibody (1:3000, #7074S, CST) for 2 h at room temperature. The final step in the process was to visualize and quantify the target proteins (ECL New England Biolabs, USA), and relative grayscale quantification was conducted with ImageJ software. The blots were cut prior to hybridization with antibodies. The original western blotting images are provided in the Supplementary Data.

### Statistical analysis

All experiments were repeated at least three times. Analyses were conducted using GraphPad Prism 9.0 software. For paired continuous variables in homologous pairing design, we conduct a normality test on the difference of paired variables using the Shapiro-Wilk test. T tests and one-way variance analyses were employed. All data are presented as the mean ± standard deviation. Statistics are considered significant when *P* ≤ 0.05.

## Results

### FSCN1 expression is elevated in OSCC

**Fig. 1:**
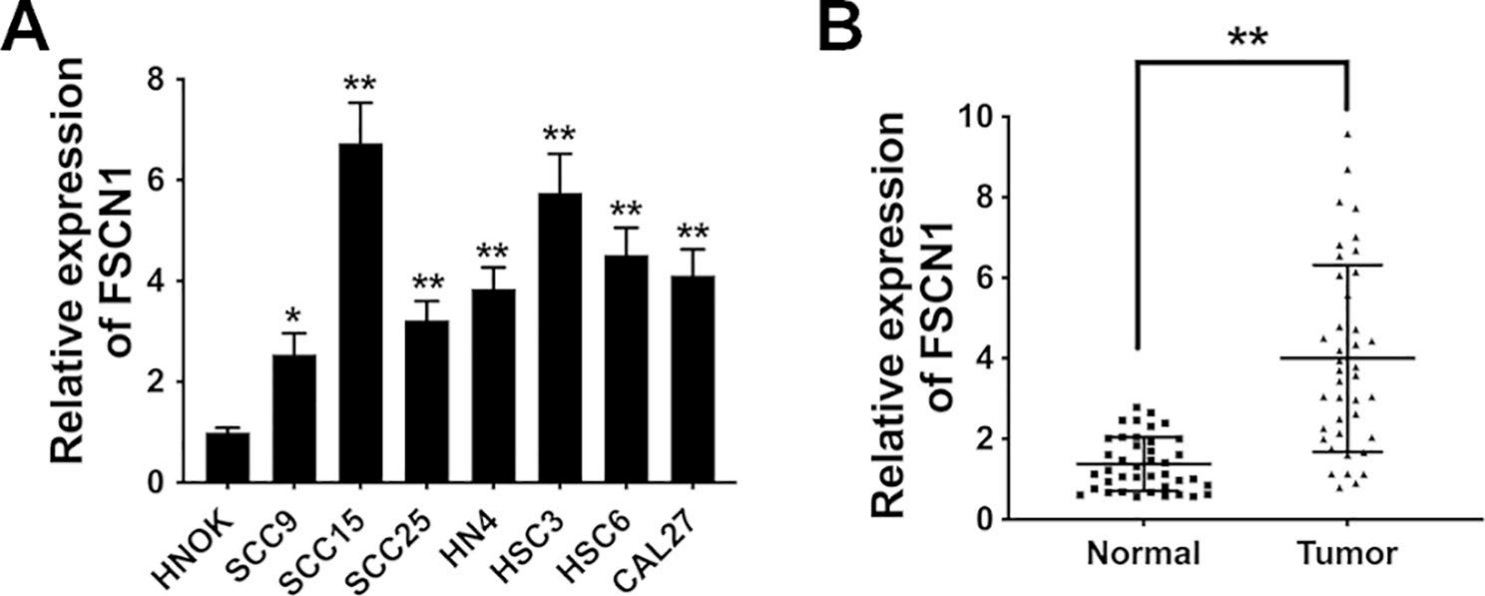
FSCN1 expression is elevated in OSCC. (**A**) FSCN1 expression in OSCC cell lines. **(B)** FSCN1 expression in 40 paired OSCC tissue specimens and neighboring noncancerous tissue specimens. Data are expressed as the mean ± SD. **P < 0.01

FSCN1 participates in the tumorigenesis process of a variety of cancers. However, the functions and underlying mechanisms of FSCN1 in OSCC remain unreported. Studies have revealed that FSCN1 is overexpressed in OSCC and correlated with survival outcomes [10, 11]. Here, qRT‒PCR assays were conducted to assess FSCN1 expression patterns in OSCC. The results showed that compared to human normal oral keratinocytes (HNOK), FSCN1 was elevated in OSCC cell lines, particularly in SCC15 and HSC3 cells (Fig. 1A). Moreover, we also verified FSCN1 expression in forty paired OSCC tissue specimens and neighboring noncancerous tissue specimens. The results revealed that the FSCN1 expression level was remarkably elevated in OSCC tissues (4.00 ± 2.29) compared to neighboring noncancerous tissue specimens (1.38 ± 0.66) (Fig. 1B).

### FSCN1 inhibition suppressed OSCC proliferation and invasion

**Fig. 2:**
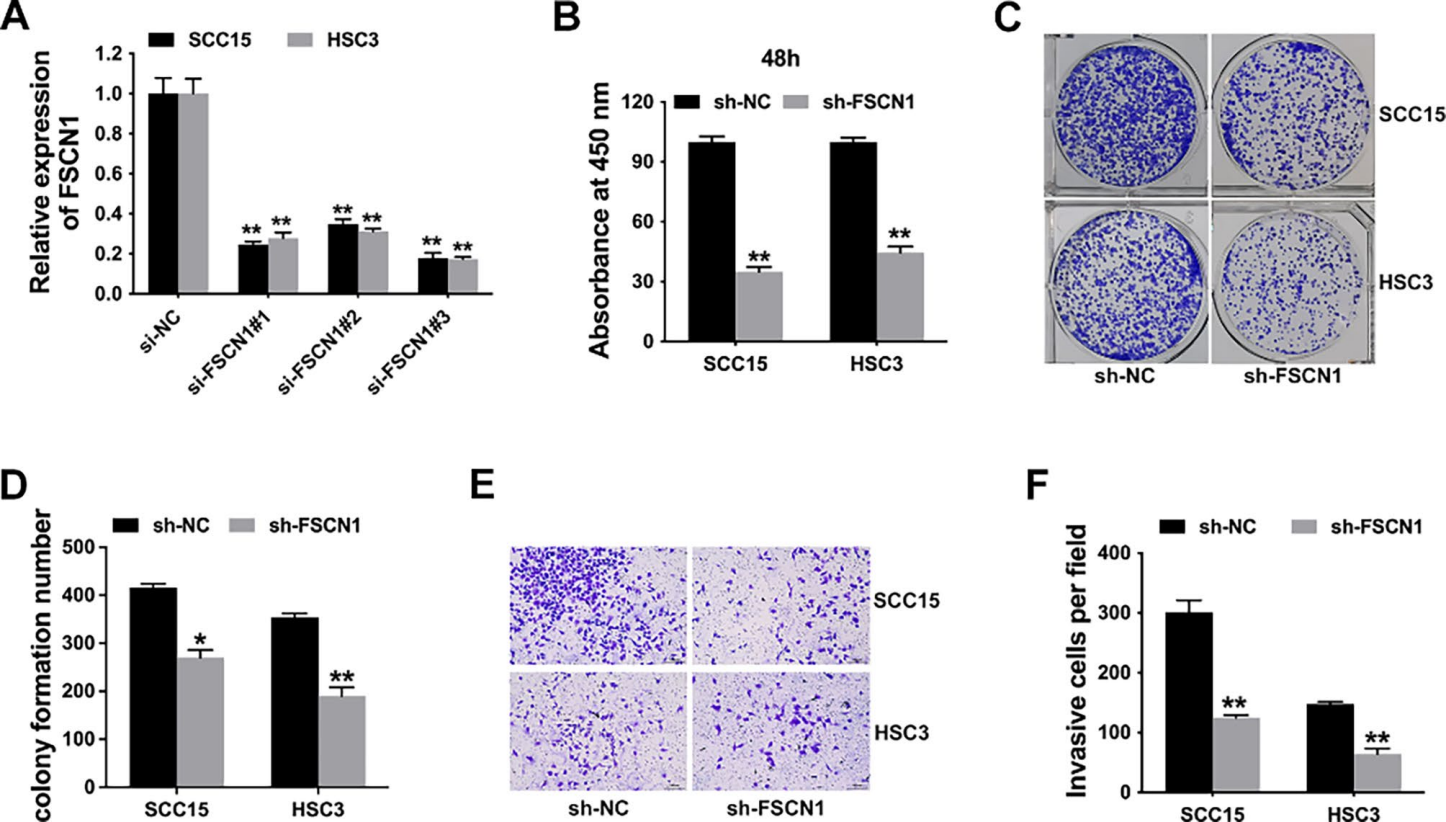
FSCN1 inhibition suppressed cell proliferation and invasion in OSCC. (**A**) The knockdown efficacy of siRNAs was detected in SCC15 and HSC3 cell lines. **(B)** CCK-8 analysis to assess the cell proliferation ability after knockdown of FSCN1 expression. **(C)** Colony formation assay was used to assess the cell proliferation ability. **(D)** Statistics for the colony formation assay. **(E)** Transwell images of SCC15 and HSC3 cell lines. **(F)** The number of invasive cells was quantified by ImageJ. Data are expressed as the mean ± SD. **P < 0.01, *P < 0.05

Due to the overexpression of FSCN1 in OSCC, we designed three siRNAs to reduce the level of FSCN1 in SCC15 and HSC3 cell lines to investigate its function. As Fig. 2A shows, FSCN1 levels were markedly reduced after transfection with si-FSCN1#3, which was used as the siRNA for further experiments. CCK-8 proliferation assays illustrated that FSCN1 inhibition dampened the proliferation capacity of OSCC cells (Fig. 2B). In addition, inhibition of FSCN1 evidently suppressed the colony formation of OSCC cells (Fig. 2C and D). Finally, transwell assays revealed that FSCN1 inhibition diminished the invasion capacity of OSCC cells (Fig. 2E F).

### Knockdown of FSCN1 suppressed OSCC cell glycolysis

**Fig. 3:**
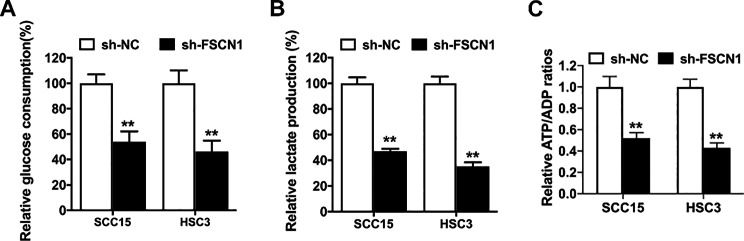
Knockdown of FSCN1 suppressed OSCC glycolysis. (**A**) Glycolytic metabolism was analyzed by glucose consumption. **(B)** Glycolytic metabolism was analyzed based on lactate production. **(C)** Glycolytic metabolism was analyzed based on the ratio of ATP/ADP. Data are expressed as the mean ± SD. **P < 0.01

Glycolysis is one of the key hallmarks of cancer and is essential for tumor growth [13]. Recently, studies have reported that glycolysis deregulation is involved in the OSCC tumorigenesis process [14]. However, the functions of FSCN1 in the OSCC glycolysis process have not been reported thus far. To assess the impact of FSCN1 on cell glycolytic metabolism in OSCC, SCC15-sh-FSCN1 cells, HSC3-sh-FSCN1 cells and control cells were subjected to glucose consumption, lactate production and ATP/ADP ratio assays. The results illustrated that FSCN1 knockdown notably decreased glucose consumption levels (Fig. 3A), lactate production levels (Fig. 3B) and the ATP/ADP ratio (Fig. 3C) in OSCC cells. All the above results revealed that downregulation of FSCN1 could suppress the glycolysis process of OSCC.

### FSCN1 regulates OSCC progression via the IRF4/Akt signaling pathway

**Fig. 4:**
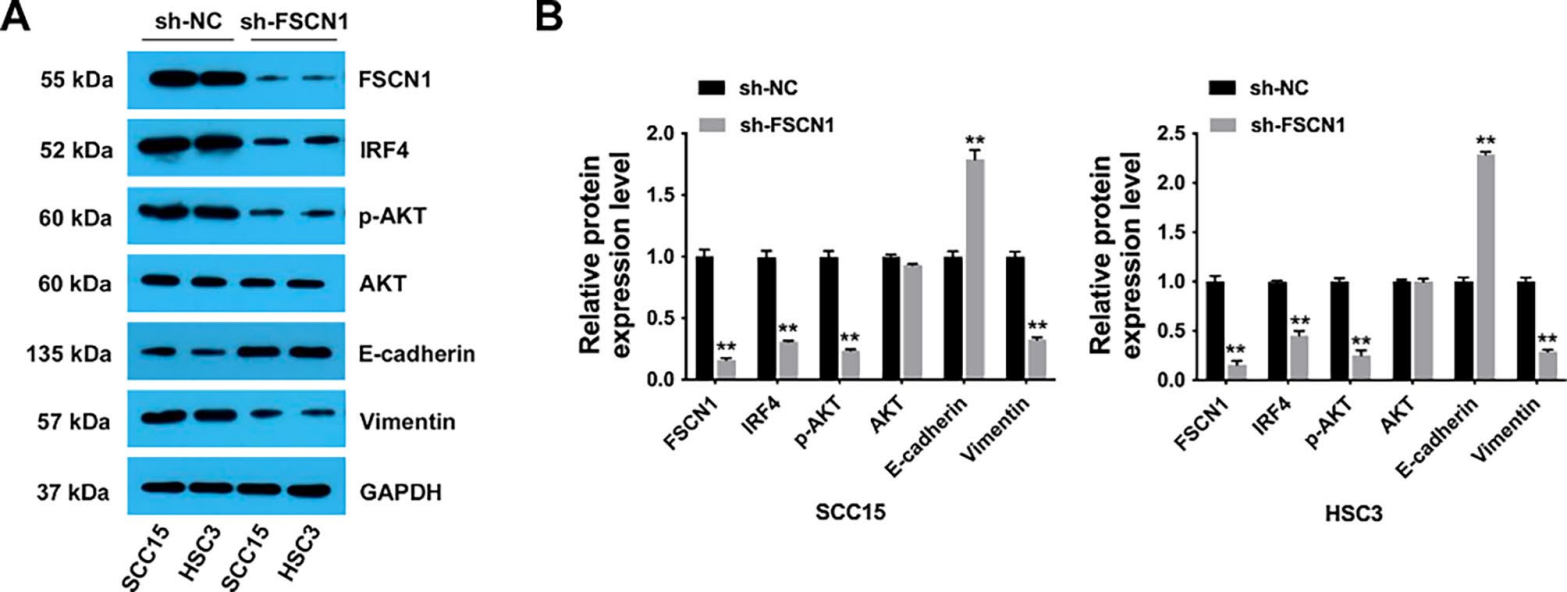
FSCN1 regulates OSCC progression via the IRF4/Akt signaling pathway. (**A**) SCC15 and HSC3 cells were subjected to western blotting assays. **(B)** The expression levels in the western blotting assay were quantified by ImageJ software. Data are expressed as the mean ± SD. **P < 0.01, *P < 0.05

Next, we continued to explore the potential mechanism of FSCN1 in the OSCC tumorigenesis process. Through bioinformatics analysis, we found that FSCN1 could interact with interferon regulatory factor 4 (IRF4), which is related to immunological disorders as well as oncogenesis [15]. For instance, in non-small cell lung cancer, IRF4 is overexpressed and enhances tumorigenesis partially through Notch-Akt signaling [16]. Thus, we detected whether FSCN1 silencing suppressed IRF4-AKT signaling activation. The results suggested that FSCN1 inhibition notably reduced the expression of IRF4 in OSCC cells (Fig. 4A and B). In addition, the phosphorylation level of AKT was reduced by FSCN1 inhibition, while the overall expression levels of AKT were not affected (Fig. 4A and B). Moreover, FSCN1 inhibition in OSCC cells obviously increased E-cadherin expression and decreased vimentin expression (Fig. 4A and B). Taken together, these results revealed that FSCN1 participates in the OSCC tumorigenesis process partly by promoting IRF4-AKT pathway activation.

## Discussion

OSCC is indeed the most common cancer in the oral cavity worldwide, and it has a poor prognosis and a high mortality rate [17]. Even with recent advances in treatments, such as targeted drugs and immunotherapy, the long-term prognosis is poor. Due to the limited treatment options for late-stage OSCC, the survival outcome is poor due to cancer metastasis, recurrence and drug resistance [18]. Hence, we need more comprehensive investigations of the mechanism of OSCC tumorigenesis to explore effective treatments to improve the outcome of OSCC.

FSCN1 is upregulated in multiple cancers and is related to cancer metastasis and a poor prognosis [19–22]. In OSCC, FSCN1 is also upregulated and associated with advanced clinical stage [23, 24]. So far, there have been only a few studies that involve the role of FSCN1 in OSCC. FSCN1 has been reported to regulate migration [25] and cell–cell contact [26] to promote OSCC progression. However, the exact mechanisms of FSCN1 deregulation and functions in OSCC are still under investigation. Further experimental verification is needed. Here, we investigated FSCN1 expression patterns in OSCC cells as well as tissues and found that OSCC cells expressed high levels of FSCN1 (Fig. 1). Subsequent functional experiments revealed that FSCN1 inhibition suppressed OSCC cell proliferation and invasion (Fig. 2).

Emerging studies have demonstrated that the molecular pathogenesis of OSCC is complex, involving dysregulated metabolites [27], which is one of the hallmarks of cancer [28]. The Warburg effect is observed in OSCC due to the requirement of a robust glucose supply to fuel cancer growth and proliferation [29, 30]. Hypoxia-related glucose metabolism, also known as glycolysis, is correlated with OSCC carcinogenesis. Inhibition of glycolysis might therefore serve as a potential adjuvant strategy in OSCC [31].

FSCN1 has been reported to increase cell glycolysis to promote tumor growth and metastasis through the YAP1-PFKFB3 axis in lung cancer [32]. However, the functions of FSCN1 in the OSCC glycolysis process have not yet been reported. Here, we showed that FSCN1 inhibition suppressed OSCC glycolysis by decreasing glucose consumption levels, lactate production levels and the ATP/ADP ratio (Fig. 3). All the above results illustrate that FSCN1 plays an essential role in the OSCC glycolysis process.

Next, we further investigated the mechanism involving FSCN1 in OSCC progression. Through bioinformatics analysis, we found that FSCN1 could interact with IRF4, which is involved in the oncogenesis of multiple cancers. For instance, IRF4 upregulation was correlated with survival outcomes in head and neck squamous carcinoma [33]. In cholangiocarcinoma, IRF4 promotes cancer proliferation as well as metastasis by regulating PI3K/AKT signaling [34]. However, no research has focused on the relationship between FSCN1 and IRF4 in OSCC thus far. Here, we revealed that FSCN1 silencing markedly decreased the IRF4 expression level in OSCC cells (Fig. 4).

In esophageal squamous cell carcinoma, FSCN1 was reported to promote tumor progression by the AKT/GSK3β signaling pathway [35]. Moreover, in lung adenocarcinoma, FSCN1 promotes tumor development through PI3K/AKT signaling [36]. Notably, in OSCC, PGK1 promotes cell glycolysis and EMT by activating AKT signaling [37]. B7-H3 promotes OSCC progression and the glycolytic metabolic program via PI3K/Akt/mTOR signaling [38]. However, the relationship between FSCN1 and AKT in OSCC has not yet been reported. In this study, we showed that FSCN1 knockdown decreased phosphorylated AKT levels, showing that FSCN1 may contribute to OSCC progression partly through an AKT-dependent pathway (Fig. 4).

In summary, our study revealed the important role that FSCN1 plays in OSCC. FSCN1 is overexpressed and acts as a promoter of OSCC proliferation, invasion, and glycolysis partially via IRF4-Akt signaling activation. Hence, it is possible that FSCN1 could be used as a treatment target as well as a biomarker for OSCC. Further research is needed to validate and optimize these findings for the future development of an FSCN1-targeted strategy for OSCC.

## Conclusions

FSCN1 expression is markedly higher in OSCC tissues than in normal tissues. FSCN1 silencing suppressed OSCC proliferation, invasion and glycolysis partially via a reduction in IRF4/AKT activation. Despite the lack of clarity regarding the detailed mechanism, this study clarified the crucial biological role played by FSCN1 during OSCC tumorigenesis. OSCC might benefit from the use of FSCN1 as both a biomarker and a target molecule.

## Electronic supplementary material

Below is the link to the electronic supplementary material.

Supplementary Material 1

## Data Availability

Authors’ data use agreement for the dataset does not permit public posting of this patient information. If anyone want to request data from this study, please contact Zhiyong Li.

## References

[1] Chen SH, Hsiao SY, Chang KY, Chang JY. New Insights into oral squamous cell carcinoma: from clinical aspects to Molecular Tumorigenesis. Int J Mol Sci. 2021;22(5). 10.3390/ijms22052252 PMC7956378 33668218

[2] Bugshan A, Farooq I (2020). Oral squamous cell carcinoma: metastasis, potentially associated malignant disorders, etiology and recent advancements in diagnosis. F1000Res.

[3] Thomson PJ (2018). Perspectives on oral squamous cell carcinoma prevention-proliferation, position, progression and prediction. J Oral Pathol Med.

[4] Li Z, Shi J, Zhang N, Zheng X, Jin Y, Wen S (2022). FSCN1 acts as a promising therapeutic target in the blockade of tumor cell motility: a review of its function, mechanism, and clinical significance. J Cancer.

[5] Hashimoto Y, Kim DJ, Adams JC (2011). The roles of fascins in health and disease. J Pathol.

[6] Liu H, Zhang Y, Li L, Cao J, Guo Y, Wu Y (2021). Fascin actin-bundling protein 1 in human cancer: promising biomarker or therapeutic target?. Mol Ther Oncolytics.

[7] Gupta I, Vranic S, Al-Thawadi H, Al Moustafa AE (2021). Fascin in Gynecological Cancers: an update of the literature. Cancers (Basel).

[8] Li J, Zhang S, Pei M, Wu L, Liu Y, Li H (2018). FSCN1 promotes epithelial-mesenchymal transition through increasing Snail1 in Ovarian Cancer cells. Cell Physiol Biochem.

[9] Zhang Y, Lu Y, Zhang C, Huang D, Wu W, Zhang Y (2018). FSCN1 increases doxorubicin resistance in hepatocellular carcinoma through promotion of epithelial-mesenchymal transition. Int J Oncol.

[10] Zhang X, Feng H, Li Z, Li D, Liu S, Huang H (2018). Application of weighted gene co-expression network analysis to identify key modules and hub genes in oral squamous cell carcinoma tumorigenesis. Onco Targets Ther.

[11] Ge Y, Li W, Ni Q, He Y, Chu J, Wei P (2019). Weighted gene Co-Expression Network Analysis identifies hub genes Associated with occurrence and prognosis of oral squamous cell carcinoma. Med Sci Monit.

[12] Liu L, Wang Y, Bai R, Yang K, Tian Z (2017). MiR-186 inhibited aerobic glycolysis in gastric cancer via HIF-1alpha regulation. Oncogenesis.

[13] Ganapathy-Kanniappan S (2018). Molecular intricacies of aerobic glycolysis in cancer: current insights into the classic metabolic phenotype. Crit Rev Biochem Mol Biol.

[14] Boschert V, Klenk N, Abt A, Janaki Raman S, Fischer M, Brands RC et al. The influence of met receptor level on HGF-Induced glycolytic reprogramming in Head and Neck squamous cell carcinoma. Int J Mol Sci. 2020;21(2). 10.3390/ijms21020471 PMC7013520 31940827

[15] Nam S, Kang K, Cha JS, Kim JW, Lee HG, Kim Y (2016). Interferon regulatory factor 4 (IRF4) controls myeloid-derived suppressor cell (MDSC) differentiation and function. J Leukoc Biol.

[16] Qian Y, Du Z, Xing Y, Zhou T, Chen T, Shi M (2017). Interferon regulatory factor 4 (IRF4) is overexpressed in human nonsmall cell lung cancer (NSCLC) and activates the notch signaling pathway. Mol Med Rep.

[17] Panarese I, Aquino G, Ronchi A, Longo F, Montella M, Cozzolino I (2019). Oral and oropharyngeal squamous cell carcinoma: prognostic and predictive parameters in the etiopathogenetic route. Expert Rev Anticancer Ther.

[18] Ling Z, Cheng B, Tao X (2021). Epithelial-to-mesenchymal transition in oral squamous cell carcinoma: Challenges and opportunities. Int J Cancer.

[19] Liu C, Gao H, Cao L, Gui S, Liu Q, Li C (2016). The role of FSCN1 in migration and invasion of pituitary adenomas. Mol Cell Endocrinol.

[20] Hanker LC, Karn T, Holtrich U, Graeser M, Becker S, Reinhard J (2013). Prognostic impact of fascin-1 (FSCN1) in epithelial ovarian cancer. Anticancer Res.

[21] Luo A, Yin Y, Li X, Xu H, Mei Q, Feng D (2015). The clinical significance of FSCN1 in non-small cell lung cancer. Biomed Pharmacother.

[22] Machesky LM, Li A, Fascin (2010). Invasive filopodia promoting metastasis. Commun Integr Biol.

[23] Routray S, Kumar R, Datta KK, Puttamallesh VN, Chatterjee A, Gowda H (2021). An integrated approach for identification of a panel of candidate genes arbitrated for invasion and metastasis in oral squamous cell carcinoma. Sci Rep.

[24] Rodrigues PC, Sawazaki-Calone I, Ervolino de Oliveira C, Soares Macedo CC, Dourado MR, Cervigne NK (2017). Fascin promotes migration and invasion and is a prognostic marker for oral squamous cell carcinoma. Oncotarget.

[25] Chen SF, Yang SF, Li JW, Nieh PC, Lin SY, Fu E (2007). Expression of fascin in oral and oropharyngeal squamous cell carcinomas has prognostic significance - a tissue microarray study of 129 cases. Histopathology.

[26] Alam H, Bhate AV, Gangadaran P, Sawant SS, Salot S, Sehgal L (2012). Fascin overexpression promotes neoplastic progression in oral squamous cell carcinoma. BMC Cancer.

[27] Vitorio JG, Duarte-Andrade FF, Dos Santos Fontes Pereira T, Fonseca FP, Amorim LSD, Martins-Chaves RR (2020). Metabolic landscape of oral squamous cell carcinoma. Metabolomics.

[28] Hanahan D (2022). Hallmarks of Cancer: New Dimensions. Cancer Discov.

[29] Botha H, Farah CS, Koo K, Cirillo N, McCullough M, Paolini R et al. The role of glucose transporters in oral squamous cell carcinoma. Biomolecules. 2021;11(8). 10.3390/biom11081070 PMC8392467 34439735

[30] Wang Y, Zhang X, Wang S, Li Z, Hu X, Yang X et al. Identification of Metabolism-Associated biomarkers for early and precise diagnosis of oral squamous cell carcinoma. Biomolecules. 2022;12(3). 10.3390/biom12030400 PMC8945702 35327590

[31] Grimm M, Cetindis M, Lehmann M, Biegner T, Munz A, Teriete P (2014). Association of cancer metabolism-related proteins with oral carcinogenesis - indications for chemoprevention and metabolic sensitizing of oral squamous cell carcinoma?. J Transl Med.

[32] Lin S, Li Y, Wang D, Huang C, Marino D, Bollt O (2021). Fascin promotes lung cancer growth and metastasis by enhancing glycolysis and PFKFB3 expression. Cancer Lett.

[33] Liu S, Wang Z (2022). Interferon Regulatory factor family genes: at the crossroads between immunity and Head and Neck squamous carcinoma. Dis Markers.

[34] Wei CX, Wong H, Xu F, Liu Z, Ran L, Jiang RD (2018). IRF4-induced upregulation of lncRNA SOX2-OT promotes cell proliferation and metastasis in cholangiocarcinoma by regulating SOX2 and PI3K/AKT signaling. Eur Rev Med Pharmacol Sci.

[35] Cai H, Wang R, Tang Z, Lu T, Cui Y (2022). FSCN1 promotes esophageal carcinoma progression through Downregulating PTK6 via its RNA-Binding protein effect. Front Pharmacol.

[36] Shi Y, Xu Y, Xu Z, Wang H, Zhang J, Wu Y (2022). TKI resistant-based prognostic immune related gene signature in LUAD, in which FSCN1 contributes to tumor progression. Cancer Lett.

[37] Zhang Y, Cai H, Liao Y, Zhu Y, Wang F, Hou J (2020). Activation of PGK1 under hypoxic conditions promotes glycolysis and increases stem celllike properties and the epithelialmesenchymal transition in oral squamous cell carcinoma cells via the AKT signalling pathway. Int J Oncol.

[38] Li Z, Liu J, Que L, Tang X (2019). The immunoregulatory protein B7-H3 promotes aerobic glycolysis in oral squamous carcinoma via PI3K/Akt/mTOR pathway. J Cancer.

