# Supplementary material for: FSCN1 promotes proliferation, invasion and glycolysis via the IRF4/AKT signaling pathway in oral squamous cell carcinoma
Source: BMC Oral Health. 2023 Jul 25;23:519. doi: 10.1186/s12903-023-03191-9 (PMC10369755; doi:10.1186/s12903-023-03191-9)
Supplement: Supplementary file 1 — Supplementary Material 1 [file 12903_2023_3191_MOESM1_ESM.pdf]

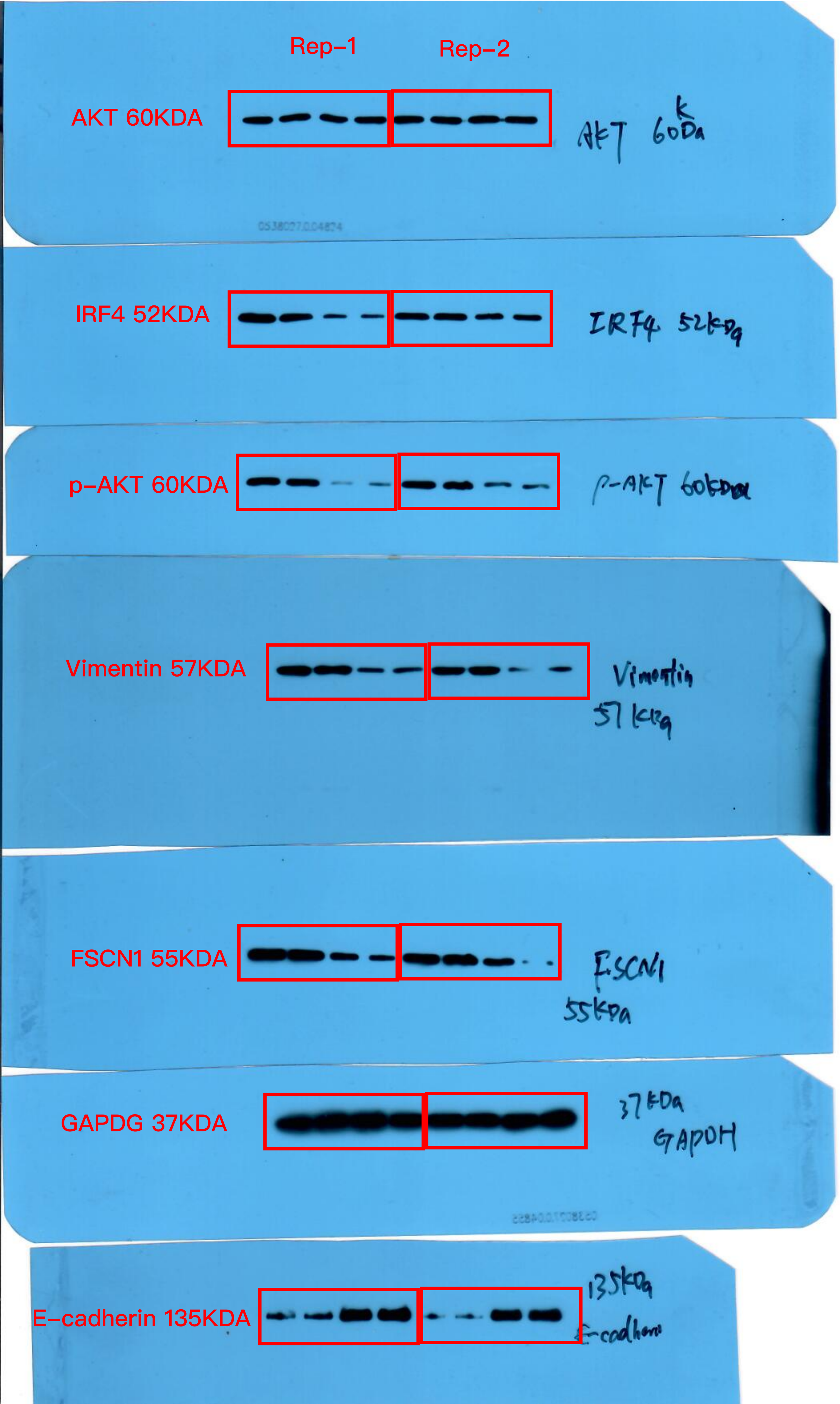
Rep-1
Rep-2
AKT 60KDA
IRF4 52KDA
p-AKT 60KDA
Vimentin 57KDA
FSCN1 55KDA
GAPDG 37KDA
E-cadherin 135KDA

E−cadherin 135KDA

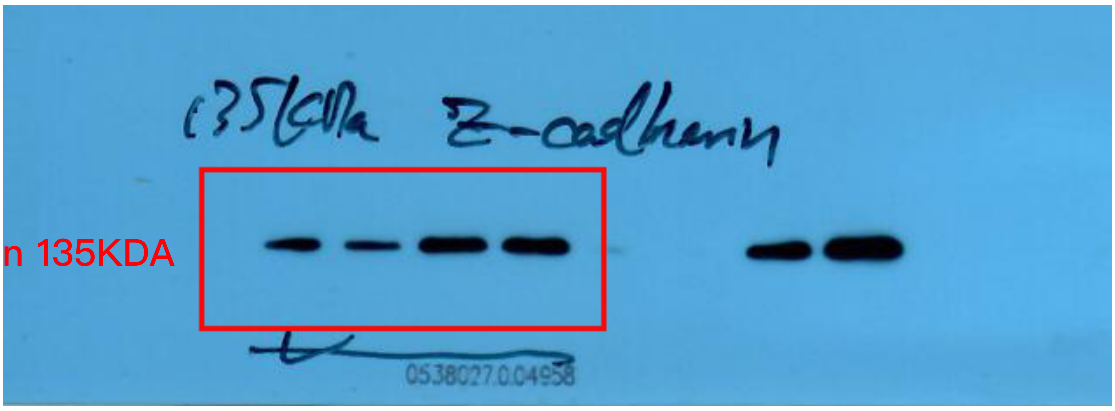

FSCN1 55KDA

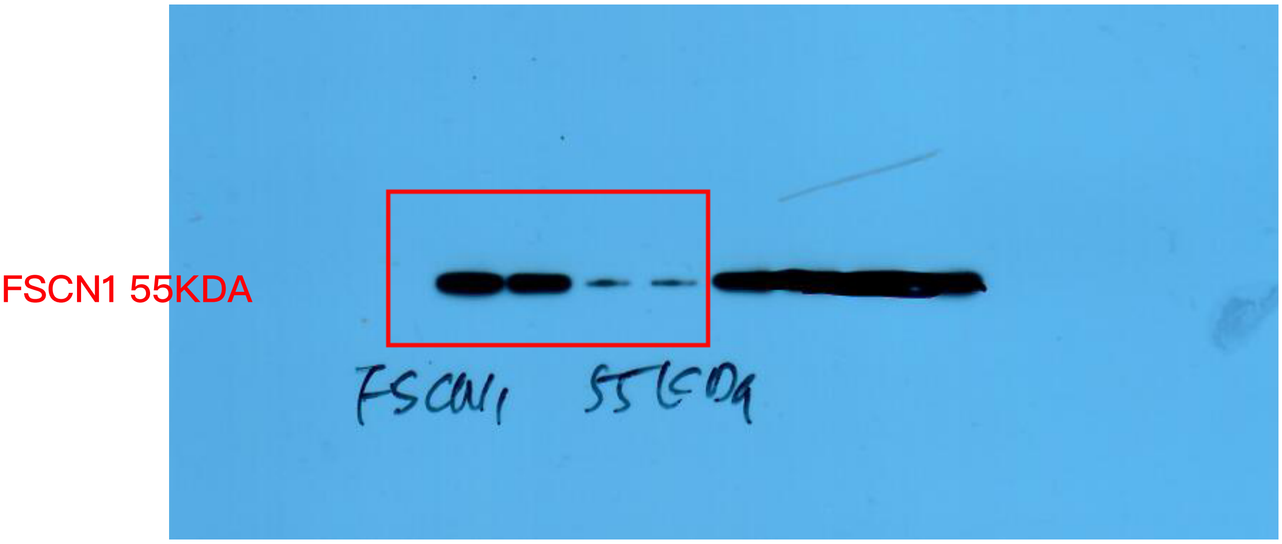

GAPDG 37KDA

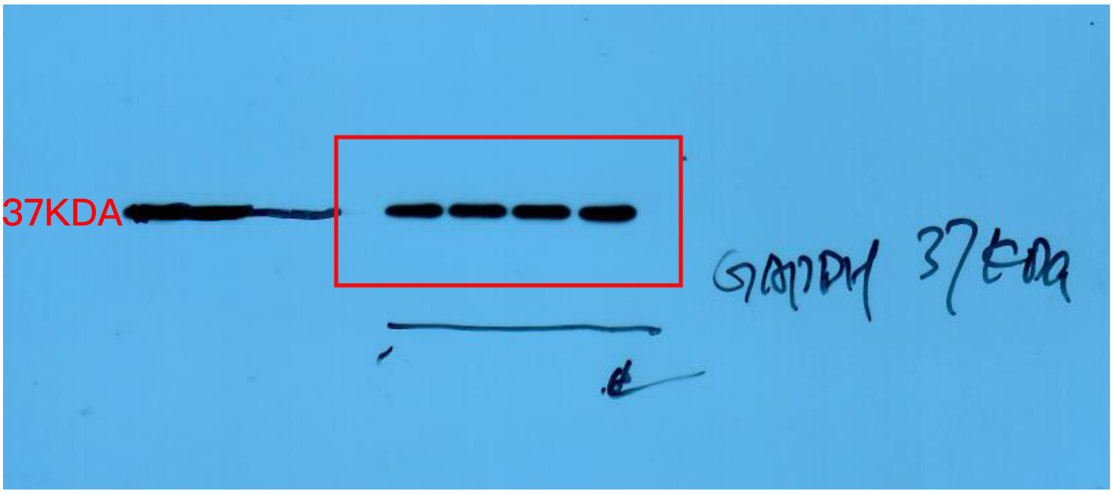

IRF4 52KDA

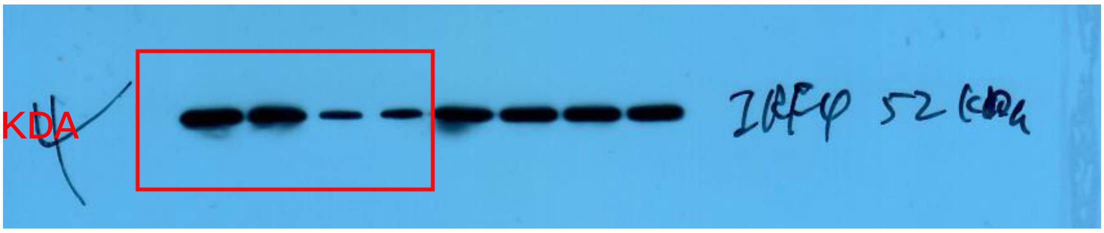

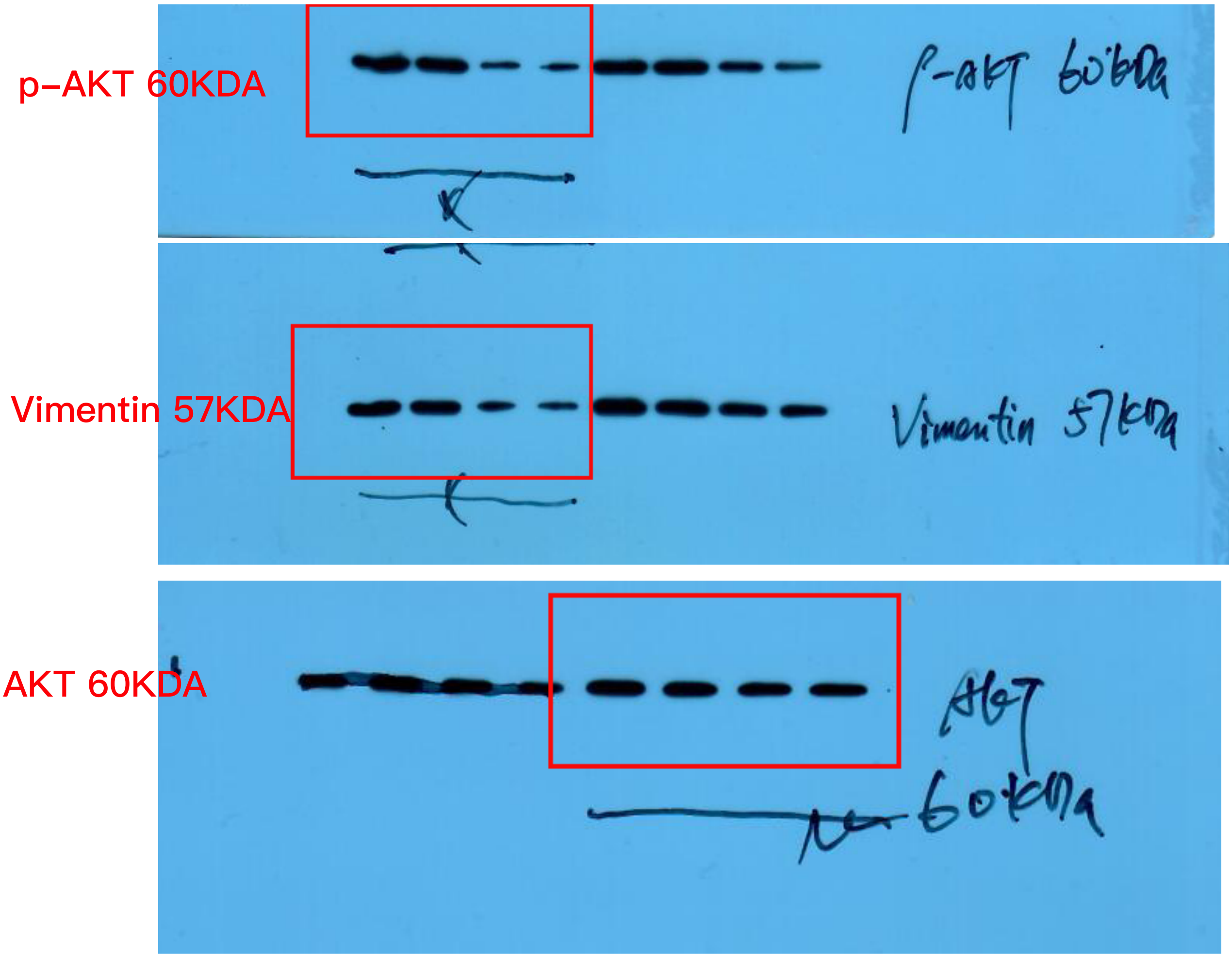

sample in each lane from left to right: **SCC15 sh-NC; HSC3 sh-NC; SCC15 sh-FSCN1; HSC3 sh-FSCN1**
